# Torsion of a Communicating Hydrocele Presented as Acute Scrotum: A Case Report and Literature Review

**DOI:** 10.1155/2016/7236104

**Published:** 2016-11-24

**Authors:** Ivonete Siviero, Ivens Baker Méio, Saulo Marcos Rebello Ferrante, Danielle Nunes Forny, André Lima da Cunha

**Affiliations:** ^1^Department of Surgery, Division of Pediatric Surgery, Medical School of Federal University of Rio de Janeiro (UFRJ), Rio de Janeiro, RJ, Brazil; ^2^Division of Pediatric Surgery, Instituto de Pediatria e Puericultura Martagão Gesteira, Federal University of Rio de Janeiro (UFRJ), Rio de Janeiro, RJ, Brazil

## Abstract

Torsion of a communicating hydrocele is extremely rare, and the cause is unclear. We report the case of a 3-year-old boy referred to us with acute scrotum. Operative findings revealed torsion of a communicating hydrocele with a 360-degree rotation of the distal end. We performed surgical excision of the necrotic cystic mass and high ligation of the peritoneal communication. A high index of suspicion is required for the correct diagnosis and treatment of this condition, which should be included among the causes of acute scrotum in childhood.

## 1. Introduction

Acute scrotum in children is usually caused by torsion of testicular appendages, testicular torsion, or epididymitis/orchitis [1]. Torsion of hernia sac or communicating hydrocele is extremely rare as a cause of acute scrotum. Only eleven cases have been reported in the literature to date [2–10]. We report the case of a 3-year-old boy with torsion of a communicating hydrocele. We describe the clinical picture and emphasize the great importance of this disease in the differential diagnosis of acute scrotum.

## 2. Case Report

**Figure 1:**
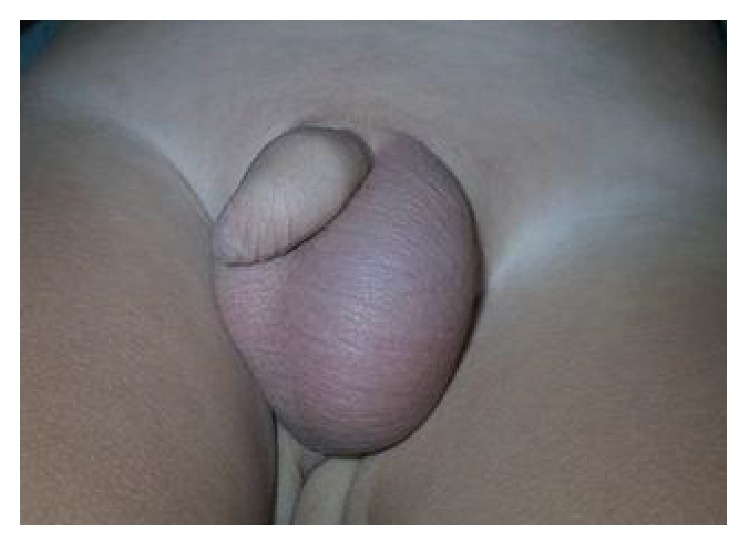
Enlargement of left hemiscrotum and a painful, palpable cystic mass.

**Figure 2:**
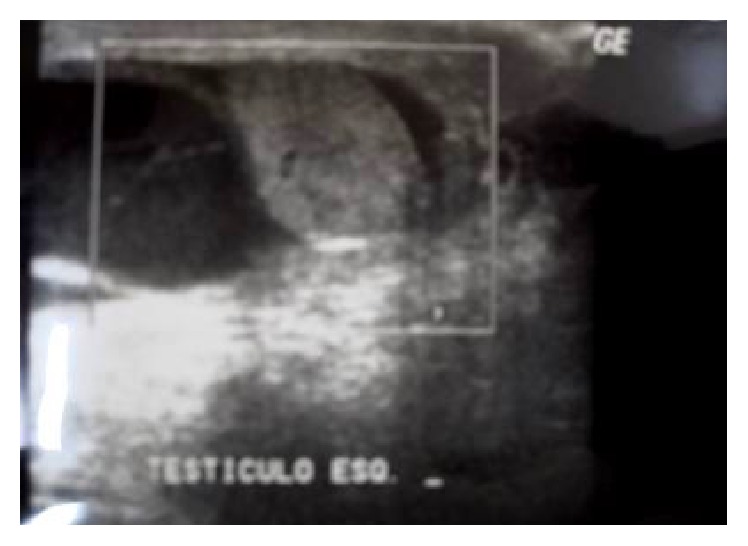
US showing a cystic mass and normal left testis.

**Figure 3:**
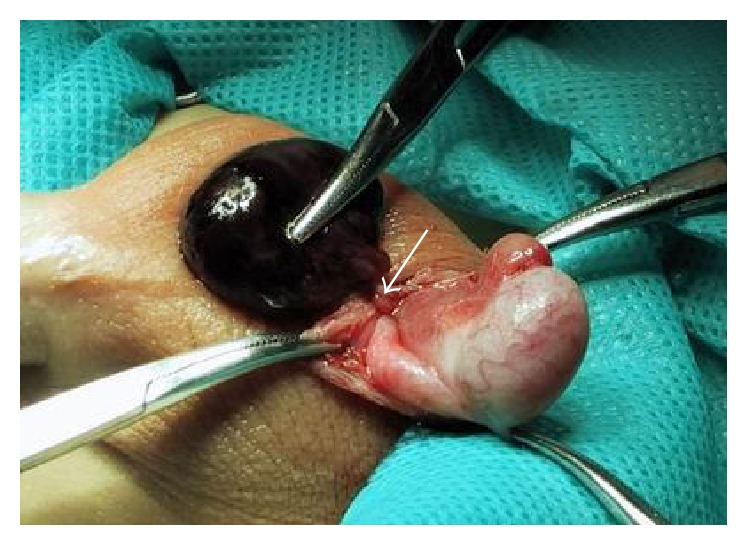
Left scrotum surgical exploration showing hydrocele filled with bloody fluid and rotated 360 degrees (arrow), with normal testis and epididymis.

A 3-year-old boy presented with pain and swelling of the left hemiscrotum lasting 13 hours. Occasionally, enlargement of the left hemiscrotum without any associated symptoms had been observed. No other symptoms, such as nausea, vomiting, or fever, were reported, nor was there a history of scrotal trauma. The left hemiscrotum was enlarged (Figure 1), without erythema, but with a palpable, tender cystic mass. The cremasteric reflex was present bilaterally. The right testis was normal. Transillumination was negative and urinalysis was normal. Ultrasonography (US) revealed an encapsulated cystic mass with heterogeneous echogenicity near the normal left testis (Figure 2). Blood flow to testis and epididymis was normal bilaterally. Emergency surgical exploration was performed. We found a hydrocele with a 360-degree rotation of the distal end, filled with bloody fluid and without communication to the testis and epididymis, both detected as normal (Figure 3). The hydrocele was resected through a scrotal approach, and peritoneal communication was repaired at the level of the internal inguinal ring through an inguinal approach. Histological examination of the cystic mass revealed greatly dilated vessels in the wall, blood infiltration, and a layer of cuboidal cells with degenerative changes. The patient was discharged 24 hours after the surgery. He was followed up every six months for a year, with no abnormalities during physical examination.

## 3. Discussion

Eleven cases of hernia sac torsion have been reported in the English literature, and only one was characterized as hydrocele torsion. Among the cases described as hernia sac torsion, two had a history of ipsilateral scrotum enlargement, suggesting communicating hydrocele. As in our case, none of these eleven patients had gastrointestinal symptoms, such as abdominal pain, nausea, or vomiting, which occur in most children with testicular torsion [11]. All patients had symptoms for a long time (more than 12 hours) and were prepubertal, with a mean age of 5 (2–10) years. Testicular torsion is more common during puberty and adolescence. Torsion of testicular appendages occurs earlier, whereas epididymitis/orchitis may occur at any age [1]. In patients with testicular torsion, the pain is usually severe and the onset is acute, but, in patients with torsion of hydrocele, hernia sac, or testicular appendages and with inflammatory diseases of testis and epididymis, the course is insidious [1].

It is difficult to unmistakably determine the correct etiology of acute scrotum. The diagnosis established on a clinical basis or through exploratory tests is not precise. Ultrasonography, particularly color Doppler imaging, continues to play a central role in the investigation of children with pain and edema of the scrotum [12]. Scintigraphy is an alternative method to assess testicular torsion and should be used when color Doppler is inadequate [12]. Magnetic resonance imaging (MRI) is more useful to evaluate abnormalities of the tunica vaginalis and testicular ischemia and infarction [12]. All cases of hernia sac or hydrocele torsion described were examined by US. Shiraishi also used MRI, which detected thickening of the spermatic fascia [7]. A US may reveal continuity of the cystic mass of the scrotum with the inguinal canal and thickening of the spermatic fascia.

The mechanism of torsion of the hernia sac or hydrocele of the scrotum is unknown. Shiraishi et al. suggested that these disorders may develop from a bell-clapper deformity, in which the hydrocele remains loose or there is slight adhesion, allowing the torsion to happen [7]. As seen during surgery, in our patient, there was no adhesion around the hydrocele and the spermatic cord, reinforcing this hypothesis. The surgical treatment consists in the closure of the peritoneal-vaginal communication and excision of the twisted cyst.

Although a conclusive preoperative diagnosis is not possible, data provided by US, coupled with history and physical findings, may point to this rare cause of acute scrotum, allowing the use of a better strategy, such as an inguinal approach, for the surgical treatment. As a result, the scrotal approach, used in most patients with acute scrotum, may be avoided.
